# Factors affecting the successful implementation of Global Polio Eradication Initiative (GPEI) in low- and middle-income countries

**DOI:** 10.7189/jogh.10.010322

**Published:** 2020-06

**Authors:** Suleiman E Mshelia, Chris Blackmore, Rachel Archer, Andrew Booth

**Affiliations:** 1 Vom Christian Hospital, Vom-Manchok Road, Jos South, Plateau State, Nigeria; 2 School of Health and Related Research (ScHARR), University of Sheffield, Sheffield, England

Polio has become the major vaccine-preventable disease that has received the much-needed support for its complete eradication following the eradication of smallpox. Currently, the incidence of the disease has been reduced by 99% owing to the intensive efforts of the Global Polio Eradication Initiative (GPEI), which was instituted in 1988 to achieve complete eradication [1]. However, the persistence of the remaining 1% of polio in endemic countries of Pakistan, Afghanistan and Nigeria has remained a concern for stakeholders and policy makers [1] The current policy of GPEI is the Polio Endgame Strategy 2019-2023 [2] which is anchored by 3 main goals: (1) Eradication; (2) Integration; (3) Certification and Containment. Implementing this policy aims to ensure that no child gets paralysed anywhere again while other public health interventions continue to benefit from the polio eradication structure. A good example of this was in 2014 when the polio vaccination infrastructure was used to halt the Ebola outbreak in Nigeria [3]. Therefore, it is imperative to develop a body of evidence regarding the factors underpinning the eradication of polio to not only support the complete eradication but also to contribute to a robust framework for curtailing other vaccine-preventable diseases.

**Figure:**
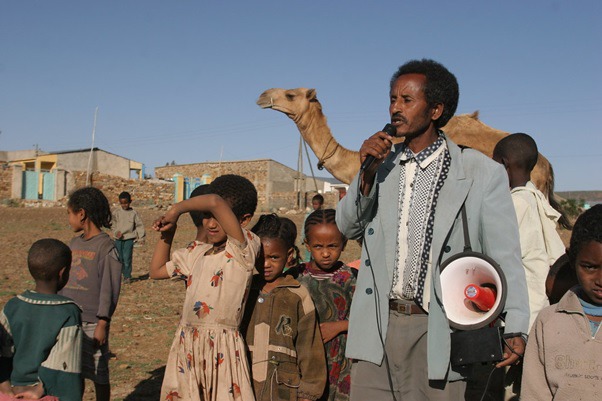
Photo: Man speaking about polio vaccination (by UNICEF Ethiopia, licensed under CC BY NC ND 2.0). Available: https://www.flickr.com/photos/86783452@N02.

The value of Qualitative Evidence Synthesis (QES) methodology in improving complex health interventions, particularly in low- and middle-income countries, is increasingly being recognised. This methodology uniquely identifies factors from qualitative research papers that can inform the improvement of health care interventions [4]. Experts argue that in-depth experiences of participants involved in any intervention are critical for the improvement of the said service [5] and this harnesses the strength of qualitative findings. In view of this, a QES regarding the successful implementation of GPEI in low- and middle-income countries was conducted by Mshelia et al. in 2019 [6], and this identified relevant barriers and facilitators regarding polio eradication.

The review used a best-fit framework of themes from Pakistan [7], which mapped themes both deductively and inductively. Generally, the findings from the review mapped well to the framework and new themes which emerged inductively include insecurity in high-risk polio areas, vaccine acceptability by caregivers, competing belief systems and cross-border polio surveillance (**Table 1**). A possible explanation of the new themes is that this review explored findings from different low- and middle-income countries and considered different contexts, which may have been different from that of Pakistan. Additionally, this review explored the perspective of caregivers, which greatly accounted for vaccine acceptability by caregivers (vaccine hesitancy). The significant rise in insurgency globally as from 2012 may possibly explain the new theme of insecurity in high-risk polio areas [8,9].

**Table 1:** Factors affecting polio eradication in developing countries [6]

Themes	Description
Program resources and logistics	Condition of cold chain in all aspects
	Skills and authority in resource allocation and human resource management
	Advocacy and communication resources
Technical aspects	Skills and training among staff at all levels in all aspects of the program
Program operation, management and organization	Availability of public health professionals and state of health service structure
	Administrative issues including:
	-Political influences and factors
	-Factors in vaccination areas and the field program
	-Immunization cards
	Issues around access (availability of staff; waiting times etc)
	Impact of positive/negative attitudes of health care staff
Monitoring, evaluation and feedback	Reporting and monitoring systems
	Decentralization of the health system
Insecurity in high-risk polio areas	Safety issues in regions where polio exists
Vaccine acceptability by caregivers	Perception of sterility induced by polio vaccine
	Literacy status of caregivers
	Influence of religion
	Influence of frequent visits
	Effectiveness of vaccination
	Influence of vaccine side effects
Competing belief systems	Eg, Islamic beliefs or traditional medicines
Influence of community stakeholders	Community leaders as determinants of community vaccination
The nature of disease	The understanding of polio as an influence
Cross-border polio surveillance	Factors that determine polio spread between neighbouring countries

It is important to note that the new themes of vaccine hesitancy and insecurity in high-risk polio areas are the current major barriers faced by polio-endemic countries of Nigeria, Pakistan and Afghanistan [10]. Therefore, it is imperative to address these community-level factors as a path to achieving a polio-free world.

Insecurity impacts on vaccination activities either resulting in the mass movement of children leading to missing vaccination, inaccessibility or polio workers being targeted by insurgents. A recent report condemned the killing of a dedicated polio worker in Pakistan while undertaking polio vaccination activities [11]. A suggestion for the use of military personnel for vaccination activities was made from the experience of insecurity in the Lake Chad region [12] but experts argued that this method should be used with caution as it can be counterproductive by deepening false perceptions regarding the vaccine [10]. However, partnership with the military was seen to improve coverage of polio vaccination among insecure and hard-to-reach areas in Angola [13]. It was also found to address some issues of vaccine hesitancy.

Further evidence demonstrates the use of community mobilisation using indigenous community members in conflict settings. This was a cluster randomised control trial that was carried out in conflict areas of Pakistan [14]. The findings buttressed the need to use local indigenous mobilisers and integrate polio vaccination as part of a maternal and child health package rather than a stand-alone specific polio campaign. This strategy significantly improved coverage in those settings. Further qualitative primary research should be undertaken to establish the relationship between insurgency and polio.

Another important community factor relates to vaccine resistance with underpinning factors as shown in **Table 1**. Even though this finding may take different forms, it buttresses the need to explore such factors contextually in areas of vaccine refusal, taking into account individual belief and value systems. This need underscores the fact that understanding the local perceptions of vaccine (not only polio) can be instrumental in mapping strategies to mitigate its refusal [15].

Nigeria is an important case study when it comes to addressing vaccine hesitance as remarkable success was achieved through the innovative use of Voluntary Community Mobilisers (VCM) [16]. These volunteers are a group of traditional and religious leaders that go around with the polio team combining the use of faith and science to educate communities. This builds trust within families concerning the vaccines, thereby increasing its uptake. Most importantly, the volunteers are well-known in the regions and thus leverage on previous trust. This is an important way to address vaccine hesitancy.

## WAY FORWARD

Immunisation is key to universal health coverage and that immunisation coverage is a valuable indicator of the strength of services delivered by any health system [17]. Since vaccination is a complex intervention, low- and middle-income countries need to adopt a broad perspective in addressing the issues influencing its success in their respective countries. Therefore, exploring the perspective of not only stakeholders but also of parents and caregivers for every given context will be instrumental to the improvement of polio eradication programmes. Stakeholder involvement will thus facilitate the delivery, and consequent success, of other vaccination programs.
